# Regulator of G‐Protein Signalling Protein AaRgs2 Negatively Regulates Appressorium‐Like Formation of *Alternaria alternata* Induced by Pear Cutin Monomer via the AaRgs2‐AaGα1‐AaAC Module

**DOI:** 10.1111/mpp.70209

**Published:** 2026-01-23

**Authors:** Miao Zhang, Yuanping Nan, Yongcai Li, Yang Bi, Dov B. Prusky

**Affiliations:** ^1^ College of Food Science and Engineering Gansu Agricultural University Lanzhou China; ^2^ Department of Postharvest Science of Fresh Produce Agricultural Research Organization, the Volcani Center Beit Dagan Israel

**Keywords:** *Alternaria alternata*, appressorium‐like formation, G protein regulatory factor, molecular mechanisms

## Abstract

Pathogenic fungi have developed complex and specific infection strategies to invade host tissues successfully. Regulator of G‐protein signalling (RGS) proteins exhibit GTPase‐accelerating protein activities and play a crucial role in the formation of infection structures in pathogenic fungi. However, specific regulatory mechanisms remain unclear. In the present study, observations of infection structure indicated that the deletion of *AaRgs2* resulted in a significant increase in spore germination. Appressorium‐like formation rate was compared to that of the wild‐type strain on a Gelbond hydrophobic membrane coated with 16‐hydroxyhexadecanoic acid or 1,16‐hexadecanediol, which are cutin monomers in pear peel. Transcriptome analysis during the appressorium‐like formation stage revealed 4124 differentially expressed genes (DEGs) that were annotated in the wild‐type strain and the Δ*AaRgs2* mutant. KEGG enrichment analysis showed that the cAMP‐PKA signalling pathway, MAPK signalling pathway, peroxisome and autophagy pathway were closely associated with appressorium‐like formation regulated by AaRgs2. Yeast two‐hybrid and bimolecular fluorescence complementation assays demonstrated a specific physical interaction between AaRgs2 and AaGα1, further confirming that AaGα1 interacted with the Pfam domain of adenylate cyclase AC. Our studies provide evidence suggesting that AaRgs2 negatively regulates appressorium‐like formation of *A. alternata* induced by pear cutin monomer via the AaRgs2‐AaGα1‐AaAC module.

## Graphical abstract

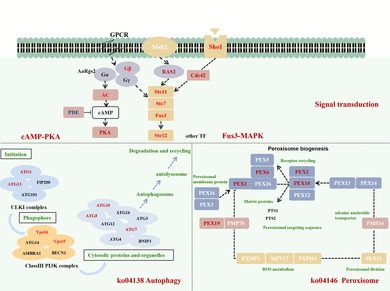

## 1 Introduction

The attachment and penetration of plant surfaces is the most important process in various plant–fungal interactions (Chethana, Jayawardena, Chen, et al. 2021). Appressoria, as the most extensively studied pathogenic fungal infection structures, play a vital role in many devastating plant diseases (Chethana, Jayawardena, Chen, et al. 2021). Numerous plant‐pathogenic fungi such as *Magnaporthe oryzae* (Hyde et al. 2020), *Colletotrichum gloeosporioides* (Wang et al. 2021), *Ustilago maydis* (Demoor et al. 2019), *Fusarium graminearum* (Mentges et al. 2020), and *Rhizoctonia* spp. (Ryder et al. 2022) have the ability to differentiate into specialised appressoria to infect the host. Certain natural components of plant cuticles, such as fatty acids, waxes and volatile compounds, are believed to act as chemical inducers of appressorium formation (Zhang et al. 2020). Spores attached to the host are stimulated by exogenous signals to germinate on the surface of the plant, forming germ tubes and finally differentiating into appressoria (Wang et al. 2021). However, the mechanism of interaction between plants and pathogenic fungi is highly intricate; understanding the molecular mechanisms of infection structure differentiation may be a new strategy for developing disease management control.

The formation of appressoria depends on G‐protein signalling to transmit exogenous stimuli to downstream cascades, initiating morphogenesis through Fus3/Kss1‐MAP kinase, cAMP‐dependent protein kinase A (cAMP‐PKA), and Target of rapamycin (TOR) signalling pathways (Marroquin‐Guzman and Wilson 2015; Jiang et al. 2018; Selvaraj et al. 2017). G‐protein mediates diverse cellular biological processes including cell growth, division and proliferation (Moretti et al. 2017). The G protein signalling regulator RGS is generally considered to serve as a GTPase activating protein to accelerate the hydrolysis of GTP in the Gα subunit to GDP, switching off the heterologous trimer G protein signalling pathways and terminating the cell process regulated by downstream signalling modules (Yan et al. 2021). Additionally, some RGS proteins can accelerate the formation of heterotrimers by enhancing the affinity of the Gα subunit for the Gβγ dimer after GTP hydrolysis (Wang et al. 2013). The regulating effect of RGS and RGS‐like proteins on the differentiation of infection structures and pathogenicity has been elucidated in various fungi such as *M. oryzae* (Zhang et al. 2011; Liu et al. 2007), *C. gloeosporioides* (Liu et al. 2018), *Fusarium verticillioides* (Mukherjee et al. 2011) and *Gibberella zeae* (Park et al. 2012). However, owing to the diverse modes of interaction between RGS and Gα subunits (Masuho et al. 2020), discrepancies exist in their regulatory roles and mechanisms across different fungi species.

*Alternaria alternata* , the causal agent of black spot or core rot, is regarded as one of the most crucial pathogens of fruit and vegetables including lemon, pear, apple, dragon fruit, citrus, tomato and cruciferous vegetables (DeMers 2022; Pan et al. 2017). Black rot of pear caused by *A. alternata* is a common postharvest disease of pears worldwide. To accomplish successful colonisation, *A. alternata* generally invades the pears by either wounds or latent infection (Li et al. 2007). It is not surprising that *A. alternata* is triggered by pear fruit epidermal signals or surface architecture to form non‐melaninised appressorium‐like structures and the infection hyphae (Tang et al. 2017). A variety of pathogenic factors are activated by the infection hyphae that invade the host epidermis through mechanical pressure (Wang et al. 2021). Previous studies have identified three RGS proteins in *A. alternata* , with findings indicating that *AaRgs2* differentially regulates the formation of appressorium‐like structures (Zhang et al. 2023), but the molecular mechanism of regulation of *AaRgs2* on formation of appressorium‐like structures in *A. alternata* remains unclear.

In this study, we examined the regulatory effect of *AaRgs2* on the formation rate of *A. alternata* infection structures on the surface of Gelbond hydrophobic membrane coated with cutin monomers 1,16‐hexadecanediol and 16‐hydroxyhexadecanoic acid, and analysed the differentially expressed genes (DEGs) between wild‐type and Δ*AaRgs2* mutant strains during the appressorium‐like formation stage using transcriptome technology. We validated the key genes involved in signalling pathways of AaRgs2 regulating *A. alternata* appressorium‐like formation through reverse transcription‐quantitative PCR (RT‐qPCR). The molecular mechanism of AaRgs2 regulating the infectious structures differentiation of *A. alternata* through the AaGα1‐AaAC module was examined by yeast two‐hybrid and bimolecular fluorescence complementation techniques. These findings establish a basis for exploring the pathogenic mechanisms of postharvest pathogens of fruits and vegetables and developing novel safe preservatives from a signal transduction perspective.

## 2 Results

### 2.1 AaRgs2 Negatively Regulates the Formation of Infection Structures in *A. alternata* Induced by Pear Cutin Monomers

**FIGURE 1:**
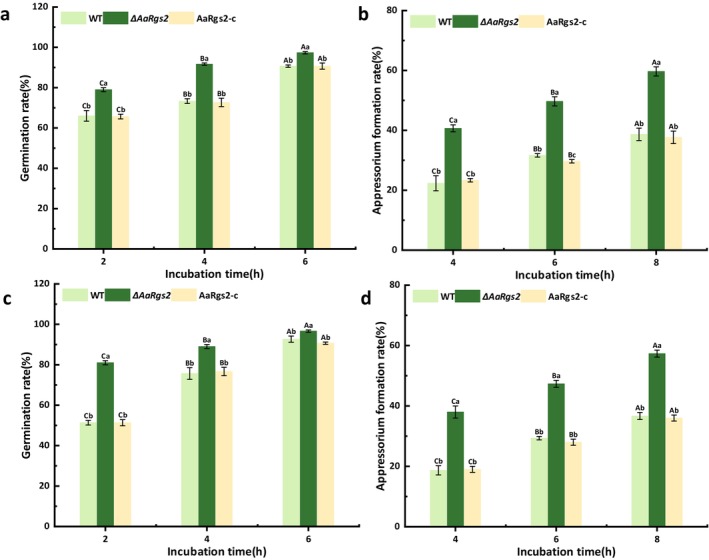
The effect of *AaRgs2* on the early infection stage of *Alternaria alternata* . The germination rates (a, c) of wild type (WT), Δ*AaRgs2* mutant and complementation strains were counted after incubation on Gelbond hydrophobic film surfaces coated with 16‐hydroxydecanoic acid (a) or 1,16‐hexadecanediol (c) for 2, 4 and 6 h. The appressorium‐like formation rates (b, d) of WT, Δ*AaRgs2* mutant and complementary strains were counted after incubation on Gelbond hydrophobic film surfaces coated with 16‐hydroxydecanoic acid (b) or 1,16‐hexadecanediol (d) for 4, 6 and 8 h. Different uppercase letters indicate significant differences between groups, and different lowercase letters indicate significant differences within groups (*p* < 0.05).

To assess the regulatory effect of *AaRgs2* on infection structure formation in *A. alternata*, the spore germination and appressorium formation rate of *A. alternata* were examined on Gelbond hydrophobic membranes coated with pear cutin monomers (1,16‐hexadecanediol and 16‐hydroxyhexadecanoic acid). The deletion of *AaRgs2* led to a significant (*p* < 0.05) increase in both spore germination and appressorium formation rates compared to the wild‐type strain, with 25.0% and 56.8% increase, respectively, after 4 h of cultivation on the surface of Gelbond hydrophobic membrane coated with 16‐hydroxyhexadecanoic acid (Figure 1a,b). Similarly, the Δ*AaRgs2* mutant strain showed a 17.6% and 62.1% increase in spore germination and appressorium formation rates, respectively, compared to the wild‐type strain after 4 h of cultivation on the surface of Gelbond hydrophobic membrane coated with 1,16‐hexadecanediol (Figure 1c,d). The defects in infection structure formation were restored in the *AaRgs2‐c* complementation strain.

The infection ability of the Δ*AaRgs2* mutant on the surface of pear fruit epidermis was assessed using a cutin transparency test. The results indicated that the appressorium‐like formation rate of the Δ*AaRgs2* mutant increased by 47% compared to the wild‐type strain after 6 h of cultivation. However, the ability of the appressorium to form infection hyphae was significantly reduced. Twelve hours after incubation, the deletion of *AaRgs2* resulted in a marked decrease in the formation rate of infection hyphae by 42.1% compared with the wild‐type strain (Figure S1).

### 2.2 Analysis of Sample Correlation and Statistics of DEGs

**FIGURE 2:**
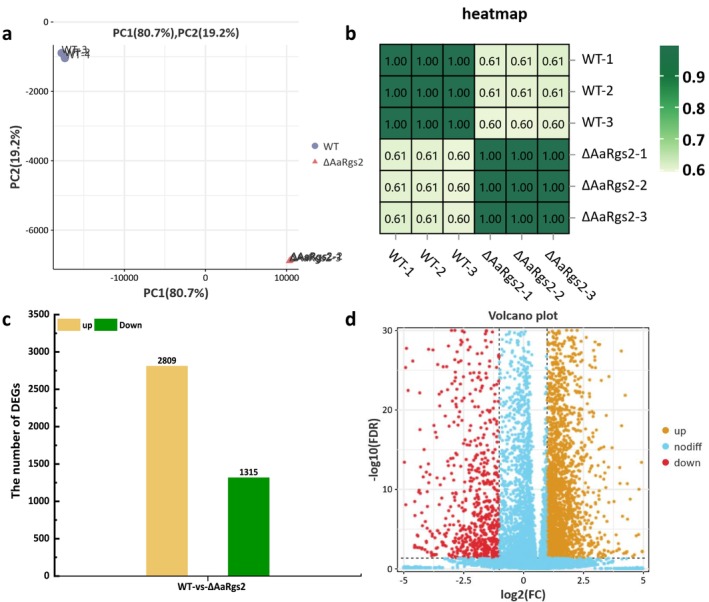
Correlation analysis between transcriptome samples of *Alternaria alternata* wild type (WT) and Δ*AaRgs2* strains grown on fruit wax‐coated Gelbond hydrophobic film (a, b). Statistics of differentially expressed genes (DEGs) between WT and Δ*AaRgs2* strains (c). Volcano plot of DEGs between WT and Δ*AaRgs2* strains (d). FC, fold change; FDR, false discovery rate.

Principal component analysis (PCA) revealed that the three biological replicates of each strain (wild type or Δ*AaRgs2*) grown on fruit wax‐coated Gelbond hydrophobic film exhibited an aggregated distribution, with a predominant proportion of variance in the first principal component (80.7%), while the second principal component accounted for only 19.2% of the variance (Figure 2a). Pearson correlation coefficients for the three biological replicates from each strain were about 1.00 (Figure 2b), indicating a high degree of correlation among the three replicates. Analysis of RNA‐seq data from wild‐type versus the Δ*AaRgs2* mutant strains identified a total of 4124 DEGs (log_2_FC > |1|, FDR < 0.05), with twice as many up‐regulated genes compared to down‐regulated genes (Figure 2c). The volcano plot displays the overall distribution of these DEGs (Figure 2d).

### 2.3 Gene Ontology Enrichment Analysis of DEGs

**FIGURE 3:**
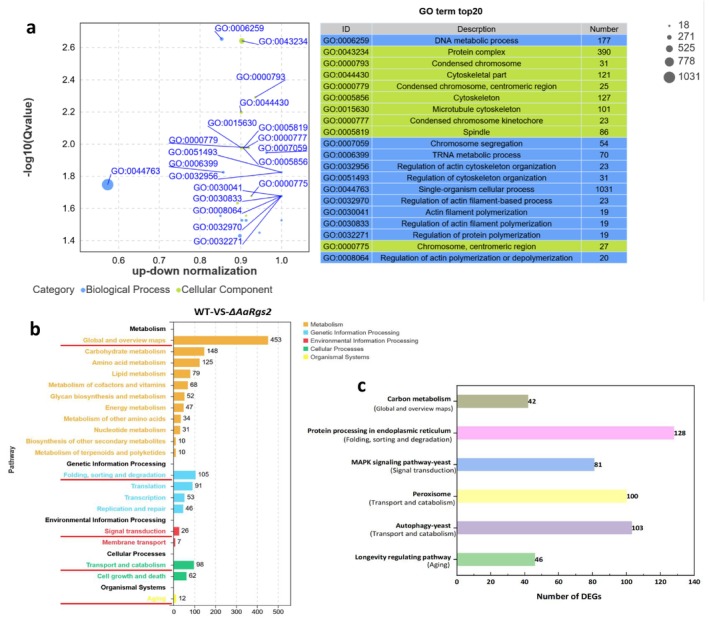
Transcriptome analysis of *Alternaria alternata* wild‐type and Δ*AaRgs2* mutant strains at the appressorium‐like formation stage. Gene Ontology enrichment analysis bubble diagram (a) and KEGG enrichment analysis histogram (b, c) of differentially expressed genes (DEGs).

Gene Ontology (GO) consists of three ontologies that describe the molecular function, cellular component and biological process of genes. The GO enrichment analysis of DEGs revealed that 47 GO terms were enriched when comparing the wild‐type strain to the Δ*AaRgs2* mutant: 21 terms associated with biological processes, 11 terms related to cellular components, and 15 terms concerning molecular functions (Figure S2). The top 20 significantly enriched GO terms in the comparison between WT and Δ*AaRgs2* mutant strains are illustrated in Figure 3a. The enriched GO terms in the cellular component category enrichment mainly consist of protein complexes, condensed chromosomes (centromeric region), cytoskeleton, and spindle. Among the 11 GO terms identified in the biological process category, the regulatory processes associated with AaRgs2 include the regulation of actin cytoskeleton organisation, actin filament‐based process, actin filament polymerisation, protein polymerisation, and actin polymerisation or depolymerisation. Additionally, three GO terms were enriched in the metabolic processes category, encompassing DNA, tRNA and single‐organism cellular metabolic processes.

### 2.4 Functional Annotation Analysis of KEGG Pathway

The identification of principal biochemical metabolic pathways and signal transduction pathways associated with DEGs can be determined through significant enrichment analysis using the KEGG Pathway database. Functional enrichment based on KEGG pathway classifies DEGs into five distinct categories: metabolism, genetic information processing, environmental information processing, cellular processes, and organismal systems. Each category can be represented by a different colour (Figure 3b). We selected five key annotation pathways: global and overview maps; folding, sorting and degradation; signal transduction; transport and catabolism; and aging. Analysis based on −log_10_ (*p*‐value) revealed that the most significantly enriched pathways for DEGs were carbon metabolism, protein processing in the endoplasmic reticulum, the MAPK signalling pathway, peroxisome, and autophagy among these five key annotation pathways (Figure 3c).

### 2.5 Analysis of the Pathways and DEGs Related to Appressorium‐Like Formation

#### 2.5.1 cAMP‐PKA Signalling Pathway

**FIGURE 4:**
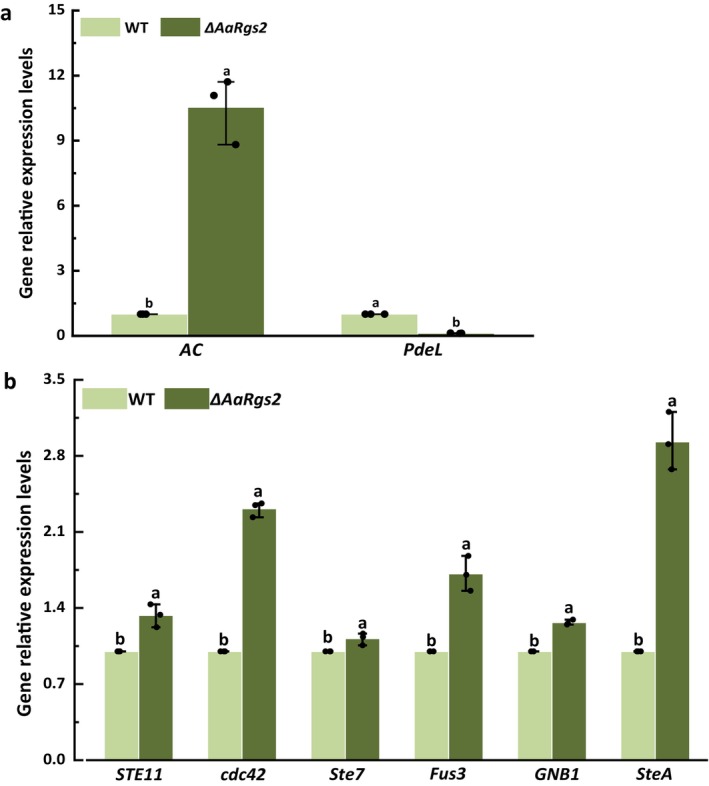
The expression levels of key genes in the cAMP‐PKA and MAPK signalling pathways in *Alternaria alternata* wild type (WT) and Δ*AaRgs2* strain at the appressorium‐like formation stage. The gene expression levels of *AC* and *PdeL* (a); the expression levels of *STE11*, *cdc42*, *Ste7*, *Fus3*, *GNB1* and *SteA* (b). The vertical line denotes the standard error (±SE). Different letters indicate significant differences (*p* < 0.05).

Two key genes in the cAMP‐PKA signalling pathway were identified among the top 10 DEGs exhibiting the most significant up‐regulation and down‐regulation. The RT‐qPCR results indicated that the expression of *AC*, a gene encoding adenylate cyclase, responsible for synthesising cAMP, was significantly up‐regulated by 9.5‐fold in the Δ*AaRgs2* mutant strain compared to the wild‐type strain (Figure 4a). Conversely, the expression level of *PdeL*, a gene encoding a phosphodiesterase that hydrolyses cAMP, was significantly down‐regulated by 88.1% in the Δ*AaRgs2* mutant strain compared with the wild‐type strain (Figure 4a). These findings validate the gene expression levels and provide compelling evidence for the role of AaRgs2 in modulating the cAMP‐PKA signalling pathway.

#### 2.5.2 Fus3‐MAPK Signalling Pathway

The Fus3‐MAPK signalling pathway plays a crucial role in regulating appressorium formation in fungi. The key genes associated with the Fus3‐MAPK signalling pathway based on log_2_FC, including *STE11*, *cdc42*, *FUZ7* (*STE7*), *Fus3*, *GNB1* and *SteA* (*Ste12*) were examined. The results of gene expression level verification were consistent with the findings from transcriptomics annotation. Compared to the wild‐type strain, the expression levels of these six key genes were significantly up‐regulated in the Δ*AaRgs2* mutant, with *STE11*, *cdc42*, *FUZ7*, *Fus3*, *GNB1* and *SteA* exhibiting up‐regulation by the factors of 1.33, 2.31, 1.12, 1.71, 1.27 and 2.93, respectively (Figure 4b).

#### 2.5.3 Autophagy Process

**FIGURE 5:**
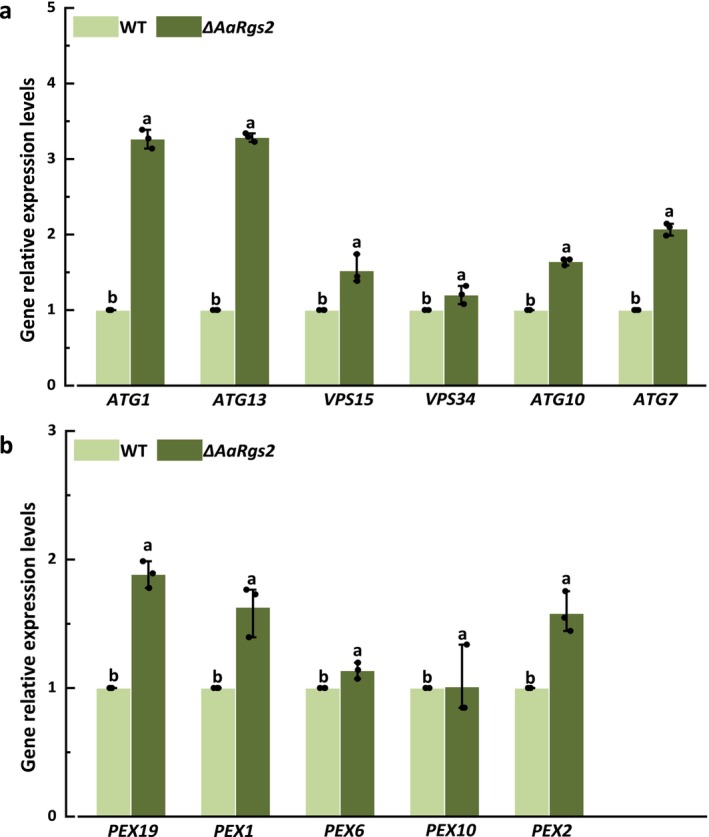
The expression levels of autophagy and peroxisome‐related genes in *Alternaria alternata* wild type (WT) and Δ*AaRgs2* strain at the appressorium‐like formation stage. The gene expression levels of *ATG1*, *ATG13*, *VPS15*, *VPA34*, *ATG10* and *ATG7* (a). The gene expression levels of *PEX19*, *PEX1*, *PEX6*, *PEX10* and *PEX2* (b). The vertical line denotes the standard error (±SE). Different letters indicate significant differences (*p* < 0.05).

The mRNA expression levels of six autophagy‐related key genes (*ATG1*, *ATG13*, *VPS15*, *VPS34*, *ATG10* and *ATG7*) were validated using RT‐qPCR. ATG1 and ATG13 are critical components of the ULK1 complex, which is responsible for the formation of phagocytic vesicles during autophagy. In the Δ*AaRgs2* mutant, the expression levels of *ATG1* and *ATG13* were up‐regulated by 3.27‐ and 3.29‐fold, respectively, compared to the wild‐type strain (Figure 5a). The Class III phosphoinositide 3‐kinase (PI3K) complex, predominantly formed by VPS15 and VPS34, plays a pivotal role in cytoplasmic protein and organelle phagocytosis. Additionally, ATG10 and ATG7 are essential for the formation of autophagosomes during the autophagy process. The expression levels of *VPS15*, *VPS34*, *ATG10* and *ATG7* were up‐regulated by 1.52, 1.20, 1.64 and 2.08‐fold, respectively, in the Δ*AaRgs2* mutant compared to the wild type (Figure 5a).

#### 2.5.4 Peroxisome Biogenesis

DEGs involved in peroxisome biogenesis are summarised in Table S1. Notably, the peroxisome‐associated genes exhibited high log_2_FC values, with five genes being validated through RT‐qPCR. The results showed that *PEX19*, *PEX1*, *PEX6*, *PEX10* and *PEX2* were significantly up‐regulated. The most striking difference in gene expression was observed for *PEX19*, which showed a 1.89‐fold up‐regulation in the Δ*AaRgs2* mutant compared to the wild‐type strain (Figure 5b). Additionally, the up‐regulation for *PEX1*, *PEX6*, *PEX10* and *PEX2* were 1.63, 1.14, 1.01 and 1.58 times, respectively (Figure 5b).

### 2.6 Molecular Mechanisms of AaRgs2 Regulation of the Appressorium‐Like Formation in *A. alternata*

#### 2.6.1 AaRgs2 Interacts With AaGα1 In Vitro and In Vivo

**FIGURE 6:**
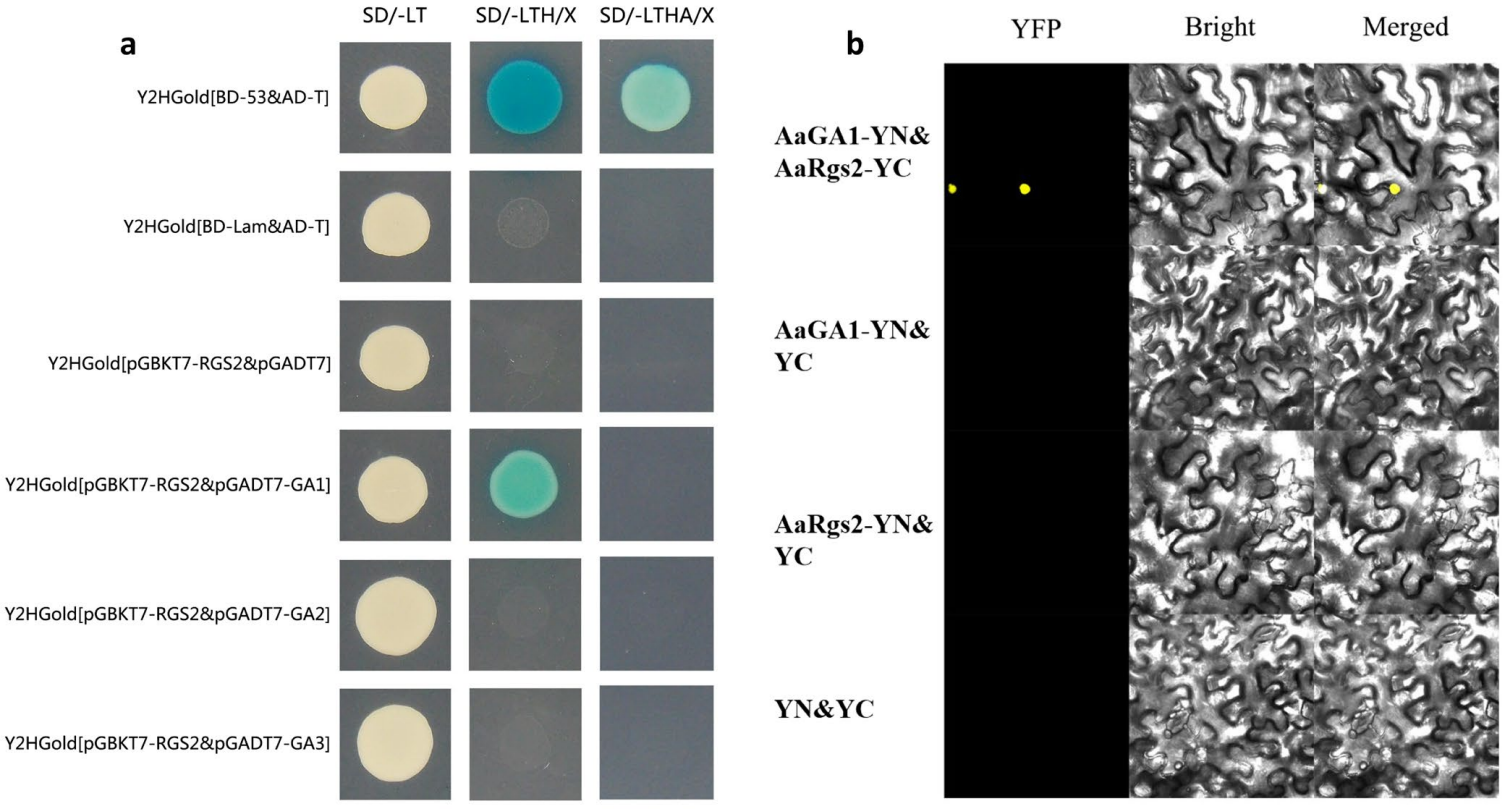
Yeast two‐hybrid assay of AaRgs2 with AaGα subunit proteins (AaGα1 [GA1], AaGα2 [GA2], AaGα3 [GA3]) (a). Bimolecular fluorescence complementation assay of AaRgs2 and AaGα1 [GA1] proteins in *Nicotiana benthamiana* leaves (b).

Three Gα subunit proteins AaGα1, AaGα2 and AaGα3 were identified in *A. alternata* through BLAST alignment and gene cloning (Figure S3). In yeast two‐hybrid (Y2H) assays, as illustrated in Figure 6a, consistent with the positive control, pGBKT7‐AaRgs2 and pGAD7‐Gα1 produced positive clones on SD−LTH medium. Y2H assays indicated a specific physical interaction between AaRgs2 and AaGα1, with no interaction observed between AaRgs2 and AaGα2 or AaGα3. The physical interaction between AaRgs2 and AaGα1 was further validated by bimolecular fluorescence complementation (BiFC) assay in *Nicotiana benthamiana* leaves, which also confirmed the nuclear localisation of both AaRgs1 and AaGα1 proteins (Figure 6b).

#### 2.6.2 Gα1 Subunit Interacts With the AC Pfam Domain

**FIGURE 7:**
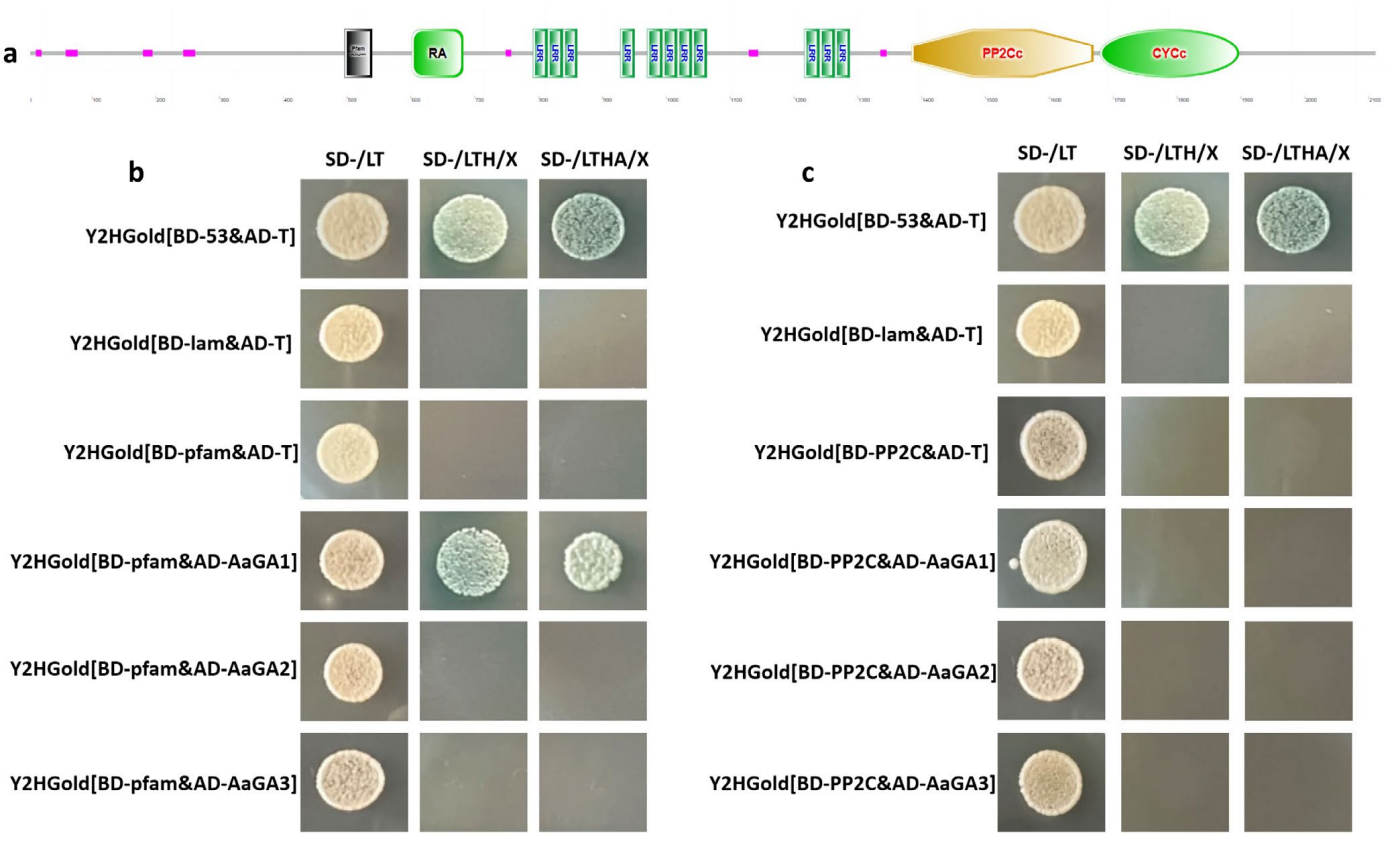
Analysis of conserved domains of AC protein (a). Yeast two‐hybrid analysis of AC protein Pfam domain (b) or PP2C domain (c) with AaGα subunit proteins (AaGα1 [GA1], AaGα2 [GA2], AaGα3 [GA3] (b)).

Because AaRgs2 and AaGα1 interacted, we also studied whether the Gα subunit proteins and AC protein interacted using Y2H assays. The AC protein is 2111 amino acids in length and contains four conserved domains: Pfam, RA, PP2C and CYC (Figure 7a). The Pfam domain catalyses the conversion of ATP to cAMP and pyrophosphate, while PP2C functions as a serine/threonine phosphate kinase responsible for phosphorylation. Interaction assays of the three Gα subunit proteins and the AC Pfam/PP2C domains revealed that AaGα1 and Pfam domain produced blue colonies on SD/−LTHA medium, indicating that AaGα1 and the Pfam domain physically interact (Figure 7b,c).

## 3 Discussion

### 3.1 AaRgs2 Negatively Regulates the Appressorium‐Like Formation and Pathogenicity of *A. alternata*

Three RGS proteins, AaRgs1, AaRgs2 and AaRgs3, were identified in *A. alternata* . We here found that AaRgs2 negatively regulated the spore germination and appressorium‐like formation of *A. alternata* induced by pear peel cutin monomer (Figure 1). This finding was consistent with the results of FvRgsA and MoRgs2, which are homologues of AaRgs2 that regulate the differentiation of infection structure in *F. verticillioides* and *M. oryzae* , respectively (Mukherjee et al. 2011; Zhang et al. 2011). Our previous studies have shown that AaRgs2, as an upstream regulator of the signalling pathway, positively regulated the pathogenicity of *A. alternata* (Zhang et al. 2023). Understanding the regulatory mechanism of infection structure formation has a positive effect on controlling diseases at the early stage. The underlying molecular mechanism by which *AaRgs2* negatively regulates the appressorium‐like formation in *A. alternata* was analysed at the RNA level in this study. Transcriptomic analysis indicated that the deletion of *AaRgs2* resulted in the up‐regulation of 2809 DEGs and the down‐regulation of 1315 DEGs (Figure 2), suggesting that AaRgs2 negatively regulates multiple biological functions. GO enrichment analysis revealed that the biological functions and cellular components influenced by AaRgs2 were primarily associated with the cytoskeleton and actin filaments, as depicted in the top 20 GO terms (Figure 3). The precise modulation of fungal microfilaments and microtubule skeletons coordinates various important mechanisms from signal perception to appressorium maturation and host colonisation (Dulal et al. 2020; Liu et al. 2022). Previous studies have shown that the actin binding protein MoAbp1 (Li, Zhang, et al. 2019) and the actin motor proteins MoMyo2 and MoMyo5 are involved in the regulation of appressorium formation in *M. oryzae* (Zheng et al. 2021). We hypothesised that the negative regulation of appressorium formation by AaRgs2 is associated with the actin microfilament cytoskeleton, but its specific regulatory mechanism remains to be elucidated. KEGG enrichment analysis revealed that the pathways significantly enriched by DEGs included metabolism, protein processing in the endoplasmic reticulum, the MAPK signalling pathway, peroxisome function, autophagy, and the longevity‐regulating pathway, as illustrated in the five key annotations (Figure 3). Previous studies have showed that the MAPK cascade signalling pathway, peroxisomes, and autophagy pathways play critical roles in the differentiation of infection structures in plant‐pathogenic fungi (Lv et al. 2022; Falter and Reumann 2022; Rogers and Egan 2020).

### 3.2 Signalling Pathways and Key Genes Involved in AaRgs2 Regulation of *A. alternata* Appressorium‐Like Formation

The key genes *AC* and *PdeL* in the cAMP‐PKA signalling pathway exhibited significant differential expression in the wild‐type and the Δ*AaRgs2* mutant strains (Figure 4). Previous studies have demonstrated that AC and PdeL play crucial roles in the regulation of appressorium formation in filamentous fungi (Yang et al. 2018). Notably, the deletion of *MAC1* leads to the termination of appressorium formation in *M. oryzae* , resulting in the inability to penetrate rice leaf cells (Choi and Dean 1997). Furthermore, MoPDEH negatively regulates appressorium formation on hydrophilic surfaces (Yang et al. 2018). Our previous research indicated that the cAMP‐PKA signalling pathway regulates the appressorium‐like formation in *A. alternata* induced by physicochemical signals from pear fruit cuticular wax (Zhang et al. 2020). Thus, it is speculated that AaRgs2 negatively regulates appressorium‐like formation through regulating the cAMP‐PKA pathway. The Fus3‐MAPK signalling pathway is the most extensively studied MAPK signalling pathway in filamentous fungi; the deletion mutant of *MrSt12* is unable to form the infection structure appressorium and is thus nonpathogenic (Meng et al. 2019). In this study, we identified key genes, including *STE11, cdc42, FUZ7, Fus3, GNB1* and *SteA*, that are significantly up‐regulated within the Fus3‐MAPK signalling pathway (Figure 4). This up‐regulation indicates that AaRgs2 negatively regulates the Fus3‐MAPK signalling pathway. The plant surface represents a nutrient‐deficient environment; thus, autophagy may supply the necessary materials and energy to support spore germination and the formation of appressorium or other infection structures (Ryder et al. 2022; Zhu et al. 2019). RT‐qPCR analysis showed that autophagy‐related genes, including *ATG1*, *ATG13*, *VPS15*, *VPS34*, *ATG10* and *ATG7*, were up‐regulated in the Δ*AaRgs2* mutant (Figure 5), indicating that the annotated DEGs were involved at all stages of autophagy. More than 32 peroxidase proteins have been found to be essential for peroxisome biogenesis and protein transport (Platta and Erdmann 2007). In this study, five genes encoding peroxidase—*PEX19*, *PEX1*, *PEX6*, *PEX10* and *PEX2*—were differentially annotated between the wild‐type strain and the Δ*AaRgs2* mutant (Figure 5). *PEX6* has been shown to positively regulate the appressorium formation in *A. alternata* (Wu et al. 2020), In *M. oryzae* , *C. lagenarium* and *C. orbiculare* , the absence of *PEX6* leads to abnormal appressoria and an inability to infect their respective host plants (Kimura et al. 2001; Wang et al. 2007). In addition, *PEX5*, *PEX7*, *PEX13*, *PEX14*, *PEX14/17* and *PEX19* are involved in the appressorium formation of *M. oryzae* . These findings demonstrate that key genes in the peroxisome pathway play a crucial role in regulating appressorium formation in pathogenic fungi.

### 3.3 AaRgs2 Negatively Regulates *A. alternata* Appressorium‐Like Formation via the AaGα1‐AaAC Module

**FIGURE 8:**
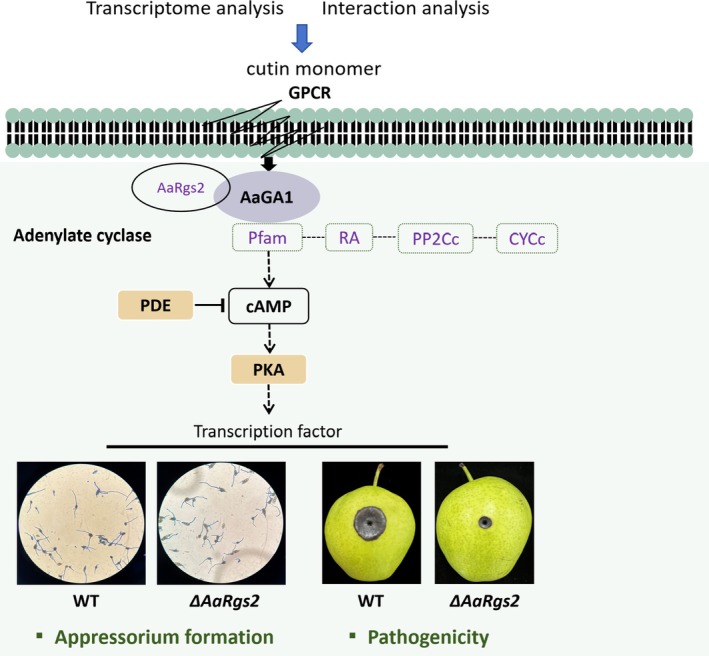
The molecular mechanism pattern of AaRgs2 regulating appressorium‐like formation in *Alternaria alternata* . Initially, the cuticular wax signal from pear fruit is received through the membrane‐sensing receptor GPCR, facilitating the dissociation of the Gαβγ complex into Gα subunits and Gβγ dimers. Subsequently, AaGα1(AaGA1) transmits signals to adenylate cyclase (AC) to synthesise the second messenger cAMP, which in turn activates the downstream PKA pathway, ultimately facilitating the formation of appressorium‐like structures. AaRgs2 plays a crucial role in expediting the repolymerisation of the Gαβγ complex. WT, wild type.

A variety of evidence has shown that G proteins are involved in the perception of nutrients (carbon and amino acids), peptide pheromones and host signals in the form of heterotrimeric complexes, thereby regulating the differentiation of infection structures and virulence in plant‐pathogenic fungi (Zhu et al. 2021). We identified three Gα subunit proteins in *A. alternata* , which correspond to the number of Gα subunit proteins found in various pathogenic fungi, including *Neurospora crassa*, *Fusarium oxysporum* and *Botrytis cinerea* (Kays et al. 2000; Guo et al. 2016; Gronover et al. 2001). The selective interaction patterns of 20 RGS proteins and 16 Gα proteins have been systematically investigated by real‐time localisation in mammals, revealing the complexity of RGS–Gα interactions (Masuho et al. 2020). The RGS–Gα interaction mode also exhibits selective differences in different plant‐pathogenic fungi. FlbA (a homologue of AaRgs1) can interact with FadA (Gα subunit) in *Aspergillus nidulans*, while RgsA (a homologue of AaRgs2) can interact with GanB (Gα subunit) (Yu 2006; Lafon et al. 2005). In *M. oryzae*, MoRGS1‐MoRGS8 proteins exhibit physical interaction with MoMAGC (Gα subunit) (Zhang et al. 2011). Among the three RGS proteins identified in *A. alternata* , only the AaRgs2 protein physically interacted with the AaGα1 subunit (Figure 6), while AaRgs1 and AaRgs3 showed no interaction with the Gα subunit (Figure S4). Previous studies indicate that AaGα1 is involved in regulating appressorium‐like formation in *A. alternata* (Nan et al. 2024). It is speculated that AaRgs2, functioning as a GTPase‐accelerating protein, promotes GTP hydrolysis of AaGα1, thereby regulating downstream signalling pathways associated with appressorium‐like formation. In *M. oryzae*, systematic studies have revealed that MoRgs1 is phosphorylated by the casein kinase MoCk2, which regulates intracellular cAMP levels, appressorium formation, and pathogenicity (Yu et al. 2021). MoRgs3 regulates appressorium formation and pathogenicity by sensing intracellular reactive oxygen species (ROS) levels (Zhang et al. 2024). Additionally, MoRgs7 acts as a receptor that participates in the cAMP pathway, playing a critical role in appressorium formation by sensing environmental hydrophobic cues (Li, Zhong, et al. 2019). In this study, transcriptome data showed that the cAMP‐PKA signalling pathway is a key pathway through which AaRgs2 regulates the formation of infection structures in *A. alternata* . Adenylate cyclase (AC) plays an important role in the growth and development, formation of infection structures and pathogenicity of fungi by synthesising cAMP and activating downstream protein kinase A (Cai et al. 2023). The dynamic balance of cAMP content is essential for the biological functions of filamentous fungi, with enzyme activity related to the synthesis and hydrolysis of intracellular cAMP levels influencing infection structure formation and pathogenicity (Yang et al. 2018). AC contains a variety of key domains. In fungi, different external signals, such as peptidoglycan, carbon dioxide, pH and temperature, can activate AC activity through distinct domains (Xu et al. 2008). In *S. cerevisiae* , Sgt1 has been shown to affect cAMP signal transduction by directly interacting with the LRR domain of Cyr1 (AC) (Dubacq et al. 2002). This study revealed that the AaGα1 protein physically interacts with the Pfam domain of AC (Figure 7). The primary function of the Pfam domain is to synthesise cAMP, while the deletion of *AaRgs2* has been shown to lead to a significant increase in cAMP content and AC enzyme activity (Zhang et al. 2023). In addition, previous studies have shown that the cAMP‐PKA signalling pathway is involved in the regulation of infection structure formation in *A. alternata* induced by physicochemical signals from pear fruit cuticular wax (Zhang et al. 2021). These results confirmed that AaRgs2 regulates the appressorium‐like formation of *A. alternata* through the AaGα1‐AC‐cAMP module. Upon the entry of the cuticular wax monomer into the cell through the G protein‐coupled receptor GPCR, AaGα1 transmits the exogenous signal to AC, resulting in the synthesis of cAMP. Subsequently, the downstream PKA pathway is activated, promoting the formation of appressorium‐like structures. As a negative regulator of AaGα1, AaRgs2 can promote the recombination of Gα subunits and Gβγ dimers into heterotrimeric complexes and hinder the transmission of exogenous signals to downstream pathways (Figure 8). However, as an upstream signalling molecule, the contribution of AaRgs2 to appressorium formation cannot be attributed to a single regulatory process and may involve multiple signalling cascades.

## 4 Experimental Procedures

### 4.1 Fungal Strains and Culture Conditions

The wild‐type strain of *Alternaria alternata* was isolated from the diseased‐healthy junction of naturally decayed pear fruit (cv. Zaosu) and characterised by sequencing the 16S rRNA. The Δ*AaRgs2* mutant strain has been previously described (Zhang et al. 2023). The fungal strains were cultured on potato dextrose agar (PDA) at 28°C for 5 days.

### 4.2 Determination of Spore Germination and Appressorium Formation Rates

#### 4.2.1 In Vitro Assays

The spore germination and appressorium‐like formation rates were determined by referring to the method of Tang et al. (2017) with slight modifications. The Gelbond hydrophobic film (Youningwei Biotechnology Co. Ltd.) was cut into a 5 × 2 cm rectangle and coated with 40 μL cutin monomers 1,16‐hexadecanediol or 16‐hydroxyhexadecanoic acid (Sigma‐Aldrich). Subsequently, 20 μL spore suspension (10^5^ conidia/mL) of wild‐type, Δ*AaRgs2* mutant and complemented strains was dropped on the surface of the treated Gelbond hydrophobic film. The infection structure formation was observed after 8 h of culture in the dark at 28°C.

#### 4.2.2 In Vivo Assays

The epidermis of Zaosu pear was sliced into pieces measuring 3 × 3 × 1 cm. A spore suspension containing 10^5^ conidia/mL of wild‐type, Δ*AaRgs2* mutant and complemented strains was applied to the slices. All pear peel samples were placed in Petri dishes lined with 3MM filter paper that had been soaked in sterile water, and incubated in darkness at 28°C for 6 and 12 h. Following incubation, the peel samples were immersed in a 3:1 (vol/vol) ethanol‐acetic acid solution for 7 h. Subsequently, the samples were transferred to filter paper moistened with a lactic acid‐glycerol‐water solution (1:1:1 vol/vol/vol) for 3 h, and the spores were stained with lactophenol cotton blue for 5 min. Each treatment was replicated three times, and the formation rates of appressoria and infection hyphae (%) were calculated.

### 4.3 Preparation of Transcriptomics Samples

Two hundred drops of 20‐μL spore suspension of wild‐type and Δ*AaRgs2* mutant strains with a final concentration of 10^6^ spores/mL were dropped in the surface of 40 μL fruit wax‐coated Gelbond hydrophobic film (Youningwei Biotechnology Co. Ltd.). The conidia were collected after 4 h of incubation at 28°C in the dark. The samples were rapidly frozen by liquid nitrogen and stored in dry ice to be sent to Guangzhou Kidio Biotechnology Co. for transcriptomic analysis. Each strain had three replicates, the sample numbers were named WT‐1, WT‐2, WT‐3, Δ*AaRgs2‐1*, Δ*AaRgs2‐2* and Δ*AaRgs2‐3*.

### 4.4 RNA Extraction and Validation of DEGs

Transcriptomic samples were treated using TRIzol reagent for total RNA extraction according to the instructions. Subsequently, 2 μg RNA was reverse transcribed to generate cDNA using a reverse transcription kit (Takara). The concentration of sample cDNA was measured by microspectrophotometer and diluted 12‐fold to be used as a template for quantitative real‐time PCR (qPCR). Pathways associated with appressorium‐like formation were analysed based on DEGs. The expression level of DEGs in the key pathways was validated by qPCR using Applied Biosystems 7500 and Step One Plus Real‐Time PCR System. *GAPDH* was selected as the internal reference gene, and each sample was repeated three times. The *C*_t_ value was obtained by analysing the amplification curve, and the relative gene expression was calculated according to the 2^−∆∆*C*t^ method (Livak and Schmittgen 2001). RT‐qPCR primers are provided in Table S1.

### 4.5 Y2H Assays

The Y2H approach (Cai et al. 2022) was used to validate interaction between the Gα subunit with AaRgs2 proteins, as well as between the Gα subunit and the domains of the AC protein. The three Gα subunits (MAGA, MAGB, MAGC) of *M. oryzae* were used to search for homologous Gα subunit sequences in *A. alternata* on the NCBI database. Primers for the Y2H assay were designed using Primer 5.0 (Table S2). We amplified the coding sequence of the bait protein using the cDNA of the wild‐type strain of *A. alternata* as a template, cloned it into the pGADT7 vector using EcoRI and XhoI restriction enzymes. Subsequently, the coding sequence of the prey protein was amplified and cloned into the pGBKT7 vector using EcoRI and BamHI restriction enzymes. About μg of successfully constructed bait vector and prey vector were co‐transfected into the Y2Hgold yeast strain. The yeast suspension was dropped on the SD−Leu−Trp, SD−Leu−Trp−His and SD−Leu−Trp−His−Ade media. The independent colonies' growth was observed after 3 days of culture.

### 4.6 BiFC Assays

To generate the constructs for BiFC assays, the full‐length cDNA fragments of *AaRgs2* and *Aa* *Gα* subunits were amplified by PCR using the cDNA of *A. alternata* wild‐type strain as template and subcloned into the pDONR221 vector via the Gateway BP Clonase II enzyme mix. Subsequently, pDONR221‐GA1 and pDONR221‐RGS2 were recombined into the YN (pEarleyGate201‐YN) and YC (pEarleyGate202‐YC) vectors through LR reaction (Yu et al. 2017). The constructs were transformed into *N. benthamiana* leaves via *Agrobacterium*‐mediated transformation (rifampicin 15 μg/mL, kanamycin 50 μg/mL). The successfully infected plants were incubated in the culture room for 2–3 days, and the fluorescence was observed under a laser scanning confocal microscope (LSM800, Zeiss).

### 4.7 Data Analysis

The data are presented as mean ± standard deviation, calculated using WPS Office 2022. Statistical analyses were performed using SPSS v. 19.0 (SPSS Inc.). One‐way ANOVA followed by Duncan's multiple range test was employed to assess significant differences among multiple groups (*p* < 0.05). Figures were generated using Origin 2021 software.

## Author Contributions

**Miao Zhang:** writing – original draft, data curation. **Yuanping Nan:** methodology. **Yongcai Li:** funding acquisition, writing – review and editing. **Yang Bi:** conceptualization, validation. **Dov B. Prusky:** validation, writing – review and editing.

## Conflicts of Interest

The authors declare no conflicts of interest.

## Supporting information


**Figure S1:** mpp70209‐sup‐0001‐FigureS1.pptx.

**Figure S2:** mpp70209‐sup‐0002‐FigureS2.pptx.

**Figure S3:** mpp70209‐sup‐0003‐FigureS3.pptx.

**Figure S4:** mpp70209‐sup‐0004‐FigureS4.pptx.

**Table S1:** mpp70209‐sup‐0005‐TableS1.docx.

**Table S2:** mpp70209‐sup‐0006‐TableS2.docx.

## Data Availability

The data that support the findings of this study have been deposited into CNGB with accession number CNP0008646; data will be made available on request.

## References

[mpp70209-bib-0001] Cai, Y. , X. Chen , P. Li , et al. 2023. “Phosphorylation Status of a Conserved Residue in the Adenylate Cyclase of *Botrytis cinerea* Is Involved in Regulating Photomorphogenesis, Circadian Rhythm, and Pathogenicity.” Frontiers in Microbiology 14: 1112584. 36876105 10.3389/fmicb.2023.1112584 PMC9975511

[mpp70209-bib-0002] Cai, Y. , X. Liu , L. Shen , et al. 2022. “Homeostasis of Cell Wall Integrity Pathway Phosphorylation Is Required for the Growth and Pathogenicity of *Magnaporthe oryzae* .” Molecular Plant Pathology 23, no. 8: 1214–1225. 35506374 10.1111/mpp.13225 PMC9276948

[mpp70209-bib-0003] Chethana, K. W. T. , R. S. Jayawardena , Y. J. Chen , et al. 2021. “Diversity and Function of Appressoria.” Pathogens 10: 746. 34204815 10.3390/pathogens10060746 PMC8231555

[mpp70209-bib-0005] Choi, W. B. , and R. A. Dean . 1997. “The Adenylate Cyclase Gene *MAC1* of *Magnaporthe grisea* Controls Appressorium Formation and Other Aspects of Growth and Development.” Plant Cell 9, no. 11: 1973–1983. 9401122 10.1105/tpc.9.11.1973 PMC157051

[mpp70209-bib-0006] DeMers, M. 2022. “ *Alternaria alternata* as Endophyte and Pathogen.” Microbiology 168, no. 3: 001153. 35348451 10.1099/mic.0.001153 PMC9558358

[mpp70209-bib-0007] Demoor, A. , P. Silar , and S. Brun . 2019. “Appressorium: The Breakthrough in Dikarya.” Journal of Fungi 5: 72. 31382649 10.3390/jof5030072 PMC6787622

[mpp70209-bib-0008] Dubacq, C. , R. Guerois , R. Courbeyrette , et al. 2002. “Sgt1p Contributes to Cyclic AMP Pathway Activity and Physically Interacts With the Adenylyl Cyclase Cyr1p/Cdc35p in Budding Yeast.” Eukaryotic Cell 1, no. 4: 568–582. 12456005 10.1128/EC.1.4.568-582.2002 PMC118006

[mpp70209-bib-0009] Dulal, N. , A. Rogers , Y. Wang , and M. Egan . 2020. “Dynamic Assembly of a Higher‐Order Septin Structure During Appressorium Morphogenesis by the Rice Blast Fungus.” Fungal Genetics and Biology 2020, no. 140: 103385. 10.1016/j.fgb.2020.103385 32305452

[mpp70209-bib-0010] Falter, C. , and S. Reumann . 2022. “The Essential Role of Fungal Peroxisomes in Plant Infection.” Molecular Plant Pathology 23: 781–794. 35001508 10.1111/mpp.13180 PMC9104257

[mpp70209-bib-0011] Gronover, C. S. , D. Kasulke , P. Tudzynski , and B. Tudzynski . 2001. “The Role of G Protein α Subunits in the Infection Process of the Gray Mold Fungus *Botrytis cinerea* .” Molecular Plant–Microbe Interactions 14: 1293–1302. 11763127 10.1094/MPMI.2001.14.11.1293

[mpp70209-bib-0012] Guo, L. , L. Yang , C. Liang , J. Wang , L. Liu , and J. S. Huang . 2016. “The G‐Protein Subunits FGA2 and FGB1 Play Distinct Roles in Development and Pathogenicity in the Banana Fungal Pathogen *Fusarium oxysporum* f. sp. *cubense* .” Physiological and Molecular Plant Pathology 93: 29–38.

[mpp70209-bib-0013] Hyde, K. D. , Y. Dong , R. Phookamsak , et al. 2020. “Fungal Diversity Notes 1151‐1276: Taxonomic and Phylogenetic Contributions on Genera and Species of Fungal Taxa.” Fungal Diversity 100: 5–277.

[mpp70209-bib-0014] Jiang, C. , X. Zhang , H. Liu , and J. R. Xu . 2018. “Mitogen‐Activated Protein Kinase Signaling in Plant Pathogenic Fungi.” PLoS Pathogens 14, no. 3: e1006875. 29543901 10.1371/journal.ppat.1006875 PMC5854419

[mpp70209-bib-0015] Kays, A. M. , P. S. Rowley , R. A. Baasiri , and K. A. Borkovich . 2000. “Regulation of Conidiation and Adenylyl Cyclase Levels by the Gα Protein GNA‐3 in *Neurospora crassa* .” Molecular and Cellular Biology 20: 7693–7705. 11003665 10.1128/mcb.20.20.7693-7705.2000 PMC86343

[mpp70209-bib-0016] Kimura, A. , Y. Takano , I. Furusawa , and T. Okuno . 2001. “Peroxisomal Metabolic Function Is Required for Appressorium‐Mediated Plant Infection by *Colletotrichum lagenarium* .” Plant Cell 13: 1945–1957. 11487704 10.1105/TPC.010084 PMC139132

[mpp70209-bib-0017] Lafon, A. , J. A. Seo , K. H. Han , J. H. Yu , and C. d'Enfert . 2005. “The Heterotrimeric G‐Protein GanB(α)‐SfaD(β)‐GpgA(γ) is a Carbon Source Sensor Involved in Early cAMP‐Dependent Germination in *Aspergillus nidulans* .” Genetics 171: 71–80. 15944355 10.1534/genetics.105.040584 PMC1456537

[mpp70209-bib-0018] Li, L. , S. Zhang , X. Liu , et al. 2019. “ *Magnaporthe oryzae* Abp1, a MoArk1 Kinase‐Interacting Actin Binding Protein, Links Actin Cytoskeleton Regulation to Growth, Endocytosis, and Pathogenesis.” Molecular Plant–Microbe Interactions 32: 437–451. 30451565 10.1094/MPMI-10-18-0281-R

[mpp70209-bib-0019] Li, X. , K. Zhong , Z. Yin , et al. 2019. “The Seven Transmembrane Domain Protein MoRgs7 Functions in Surface Perception and Undergoes Coronin MoCrn1‐Dependent Endocytosis in Complex With Gα Subunit MoMagA to Promote cAMP Signaling and Appressorium Formation in *Magnaporthe oryzae* .” PLoS Pathogens 15, no. 2: e1007382. 30802274 10.1371/journal.ppat.1007382 PMC6405168

[mpp70209-bib-0020] Li, Y. C. , Y. Bi , and L. Z. An . 2007. “Occurrence and Latent Infection of Alternaria Rot of Pingguoli Pear (*Pyrus bretschneideri* Rehd. cv. Pingguoli) Fruit in Gansu, China.” Journal of Phytopathology 155, no. 1: 56–60.

[mpp70209-bib-0021] Liu, H. , A. Suresh , F. S. Willard , D. P. Siderovski , S. Lu , and N. I. Naqvi . 2007. “Rgs1 Regulates Multiple Gα Subunits in *Magnaporthe* Pathogenesis, Asexual Growth and Thigmotropism.” EMBO Journal 26: 690–700. 17255942 10.1038/sj.emboj.7601536 PMC1794393

[mpp70209-bib-0022] Liu, N. , W. Wang , C. He , H. Luo , B. An , and Q. Wang . 2022. “NADPH Oxidases Play a Role in Pathogenicity via the Regulation of F‐Actin Organization in *Colletotrichum gloeosporioides* .” Frontiers in Cellular and Infection Microbiology 12: 845133. 35782153 10.3389/fcimb.2022.845133 PMC9240266

[mpp70209-bib-0023] Liu, Z. Q. , M. L. Wu , Z. J. Ke , W. B. Liu , and X. Y. Li . 2018. “Functional Analysis of a Regulator of G‐Protein Signaling CgRGS1 in the Rubber Tree Anthracnose Fungus *Colletotrichum gloeosporioides* .” Archives of Microbiology 200: 391–400. 29177869 10.1007/s00203-017-1455-1

[mpp70209-bib-0024] Livak, K. J. , and T. D. Schmittgen . 2001. “Analysis of Relative Gene Expression Data Using Realtime Quantitative PCR and the 2(−ΔΔC(T)) Method.” Methods 25: 402–408. 11846609 10.1006/meth.2001.1262

[mpp70209-bib-0025] Lv, W. , Y. Xiao , Z. Xu , H. Jiang , Q. Tong , and Z. Wang . 2022. “The Paxillin MoPax1 Activates Mitogen‐Activated Protein (MAP) Kinase Signaling Pathways and Autophagy Through MAP Kinase Activator MoMka1 During Appressorium‐Mediated Plant Infection by the Rice Blast Fungus *Magnaporthe oryzae* .” mBio 13, no. 6: e0221822. 36314807 10.1128/mbio.02218-22 PMC9765475

[mpp70209-bib-0026] Marroquin‐Guzman, M. , and R. A. Wilson . 2015. “GATA‐Dependent Glutaminolysis Drives Appressorium Formation in *Magnaporthe oryzae* by Suppressing TOR Inhibition of cAMP/PKA Signaling.” PLoS Pathogens 11, no. 4: e1004851. 25901357 10.1371/journal.ppat.1004851 PMC4406744

[mpp70209-bib-0027] Masuho, I. , S. Balaji , B. S. Muntean , et al. 2020. “A Global Map of G Protein Signaling Regulation by RGS Proteins.” Cell 183, no. 2: 503–521. 33007266 10.1016/j.cell.2020.08.052 PMC7572916

[mpp70209-bib-0028] Meng, Y. , X. Zhang , N. Guo , and W. Fang . 2019. “ *MrSt12* Implicated in the Regulation of Transcription Factor AFTF1 by Fus3‐MAPK During Cuticle Penetration by the Entomopathogenic Fungus *Metarhizium robertsii* .” Fungal Genetics and Biology 131: 103244. 31228645 10.1016/j.fgb.2019.103244

[mpp70209-bib-0029] Mentges, M. , A. Glasenapp , M. Boenisch , et al. 2020. “Infection Cushions of *Fusarium graminearum* Are Fungal Arsenals for Wheat Infection.” Molecular Plant Pathology 21: 1070–1087. 32573086 10.1111/mpp.12960 PMC7368127

[mpp70209-bib-0030] Moretti, M. , L. Wang , P. Grognet , D. Lanver , H. Link , and R. Kahmann . 2017. “Three Regulators of G Protein Signaling Differentially Affect Mating, Morphology and Virulence in the Smut Fungus *Ustilago maydis* .” Molecular Microbiology 105: 901–921. 28686341 10.1111/mmi.13745

[mpp70209-bib-0031] Mukherjee, M. , J. E. Kim , Y. S. Park , M. V. Kolomiets , and W. B. Shim . 2011. “Regulators of G Protein Signalling in *Fusarium verticillioides* Mediate Differential Host–Pathogen Responses on Nonviable Versus Viable Maize Kernels.” Molecular Plant Pathology 12: 479–491. 21535353 10.1111/j.1364-3703.2010.00686.x PMC6640359

[mpp70209-bib-0032] Nan, Y. P. , M. Zhang , Y. C. Li , and Y. Bi . 2024. “The G‐Protein α Subunit AaGA1 Positively Regulates Vegetative Growth, Appressorium‐Like Formation, and Pathogenicity in *Alternaria alternata* .” Journal of Applied Microbiology 135, no. 8: lxae198. 39104199 10.1093/jambio/lxae198

[mpp70209-bib-0033] Pan, T. T. , H. B. Pu , and D. W. Sun . 2017. “Insights Into the Changes in Chemical Compositions of the Cell Wall of Pear Fruit Infected by *Alternaria alternata* With Confocal Raman Microspectroscopy.” Postharvest Biology and Technology 132: 119–129.

[mpp70209-bib-0034] Park, A. R. , A. R. Cho , J. A. Seo , et al. 2012. “Functional Analyses of Regulators of G Protein Signaling in *Gibberella zeae* .” Fungal Genetics and Biology 49: 511–520. 22634273 10.1016/j.fgb.2012.05.006

[mpp70209-bib-0035] Platta, H. W. , and R. Erdmann . 2007. “Peroxisomal Dynamics.” Trends in Cell Biology 17: 474–484. 17913497 10.1016/j.tcb.2007.06.009

[mpp70209-bib-0036] Rogers, A. M. , and M. J. Egan . 2020. “Autophagy Machinery Promotes the Chaperone‐Mediated Formation and Compartmentalization of Protein Aggregates During Appressorium Development by the Rice Blast Fungus.” Molecular Biology of the Cell 31, no. 21: 2298–2305. 32816646 10.1091/mbc.E20-01-0068 PMC7851963

[mpp70209-bib-0037] Ryder, L. S. , N. Cruz‐Mireles , C. Molinari , I. Eisermann , A. B. Eseola , and N. J. Talbot . 2022. “The Appressorium at a Glance.” Journal of Cell Science 135, no. 14: jcs259857. 35856284 10.1242/jcs.259857

[mpp70209-bib-0039] Selvaraj, P. , F. T. Hong , R. Ramanujam , and N. I. Naqvi . 2017. “Subcellular Compartmentation, Interdependency and Dynamics of the Cyclic AMP‐Dependent PKA Subunits During Pathogenic Differentiation in Rice Blast.” Molecular Microbiology 105: 484–504. 28544028 10.1111/mmi.13713

[mpp70209-bib-0040] Tang, Y. , Y. C. Li , Y. Bi , and Y. Wang . 2017. “Role of Pear Fruit Cuticular Wax and Surface Hydrophobicity in Regulating the Prepenetration Phase of *Alternaria alternata* Infection.” Journal of Phytopathology 165: 313–322.

[mpp70209-bib-0041] Wang, X. L. , D. X. Lu , and C. M. Tian . 2021. “Mucin Msb2 Cooperates With the Transmembrane Protein Sho1 in Various Plant Surface Signal Sensing and Pathogenic Processes in the Poplar Anthracnose Fungus *Colletotrichum gloeosporioides* .” Molecular Plant Pathology 22: 1553–1573. 34414655 10.1111/mpp.13126 PMC8578833

[mpp70209-bib-0042] Wang, Y. C. , Z. Y. Geng , D. W. Jiang , et al. 2013. “Characterizations and Functions of Regulator of G Protein Signaling (RGS) in Fungi.” Applied Microbiology and Biotechnology 97: 7977–7987. 23917634 10.1007/s00253-013-5133-1

[mpp70209-bib-0043] Wang, Z. Y. , D. M. Soanes , M. J. Kershaw , and N. J. Talbot . 2007. “Functional Analysis of Lipid Metabolism in *Magnaporthe grisea* Reveals a Requirement for Peroxisomal Fatty Acid β‐Oxidation During Appressorium‐Mediated Plant Infection.” Molecular Plant–Microbe Interactions 20: 475–491. 17506326 10.1094/MPMI-20-5-0475

[mpp70209-bib-0044] Wu, P. C. , C. W. Chen , C. Y. L. Choo , Y. K. Chen , J. I. Yago , and K. R. Chung . 2020. “Proper Functions of Peroxisomes Are Vital for Pathogenesis of Citrus Brown Spot Disease Caused by *Alternaria alternata* .” Journal of Fungi 6, no. 4: 248. 33114679 10.3390/jof6040248 PMC7712655

[mpp70209-bib-0045] Xu, X. L. , R. T. Lee , H. M. Fang , et al. 2008. “Bacterial Peptidoglycan Triggers *Candida albicans* Hyphal Growth by Directly Activating the Adenylyl Cyclase *Cyr1p* .” Cell Host & Microbe 4, no. 1: 28–39. 18621008 10.1016/j.chom.2008.05.014

[mpp70209-bib-0046] Yan, H. J. , Z. H. Zhou , and W. B. Shim . 2021. “Two Regulators of G‐Protein Signaling (RGS) Proteins FlbA1 and FlbA2 Differentially Regulate Fumonisin B_1_ Biosynthesis in *Fusarium verticillioides* .” Current Genetics 67: 305–315. 33392742 10.1007/s00294-020-01140-5

[mpp70209-bib-0047] Yang, L. N. , Z. Yin , X. Zhang , et al. 2018. “New Findings on Phosphodiesterases, MoPdeH and MoPdeL, in *Magnaporthe oryzae* Revealed by Structural Analysis.” Molecular Plant Pathology 19, no. 5: 1061–1074. 28752677 10.1111/mpp.12586 PMC6638029

[mpp70209-bib-0048] Yu, C. W. , R. Tai , S. C. Wang , et al. 2017. “Histone Deacetylase6 Acts in Concert With Histone Methyltransferases SUVH4, SUVH5, and SUVH6 to Regulate Transposon Silencing.” Plant Cell 29, no. 8: 1970–1983. 28778955 10.1105/tpc.16.00570 PMC5590490

[mpp70209-bib-0049] Yu, J. H. 2006. “Heterotrimeric G Protein Signaling and RGSs in *Aspergillus nidulans* .” Journal of Microbiology 44: 145–154. 16728950

[mpp70209-bib-0050] Yu, R. , X. Shen , M. Liu , et al. 2021. “The Rice Blast Fungus MoRgs1 Functioning in cAMP Signaling and Pathogenicity Is Regulated by Casein Kinase MoCk2 Phosphorylation and Modulated by Membrane Protein MoEmc2.” PLoS Pathogens 17: e1009657. 34133468 10.1371/journal.ppat.1009657 PMC8208561

[mpp70209-bib-0051] Zhang, H. , W. Tang , K. Liu , et al. 2011. “Eight RGS and RGS‐Like Proteins Orchestrate Growth, Differentiation, and Pathogenicity of *Magnaporthe oryzae* .” PLoS Pathogens 7: e1002450. 22241981 10.1371/journal.ppat.1002450 PMC3248559

[mpp70209-bib-0052] Zhang, M. , Y. Li , L. Li , et al. 2023. “AaRgs1 and AaRgs2 Differential Regulate Fungal Development, Stress Response and Appressorium‐Like Formation in *Alternaria alternata* .” Postharvest Biology and Technology 205: 112537.

[mpp70209-bib-0053] Zhang, M. , Y. C. Li , T. L. Wang , et al. 2021. “AaPKAc Regulates Differentiation of Infection Structures Induced by Physicochemical Signals From Pear Fruit Cuticular Wax, Secondary Metabolism, and Pathogenicity of *Alternaria alternata* .” Frontiers in Plant Science 12: 642601. 33968101 10.3389/fpls.2021.642601 PMC8096925

[mpp70209-bib-0054] Zhang, R. , X. Liu , J. Xu , et al. 2024. “MoRgs3 Functions in Intracellular Reactive Oxygen Species Perception‐Integrated cAMP Signaling to Promote Appressorium Formation in *Magnaporthe oryzae* .” mBio 15, no. 8: e0099624. 38980036 10.1128/mbio.00996-24 PMC11323498

[mpp70209-bib-0055] Zhang, Y. L. , C. X. You , Y. Y. Li , and Y. J. Hao . 2020. “Advances in Biosynthesis, Regulation, and Function of Apple Cuticular Wax.” Frontiers in Plant Science 11: 1165. 32849720 10.3389/fpls.2020.01165 PMC7419609

[mpp70209-bib-0056] Zheng, C. , W. Zhang , S. Zhang , G. Yang , L. Tan , and M. Guo . 2021. “Class I Myosin Mediated Endocytosis and Polarization Growth Is Essential for Pathogenicity of *Magnaporthe oryzae* .” Applied Microbiology and Biotechnology 105: 7395–7410. 34536105 10.1007/s00253-021-11573-8

[mpp70209-bib-0057] Zhu, P. , S. Zhang , R. Li , et al. 2021. “Host‐Induced Gene Silencing of a G Protein α Subunit Gene CsGpa1 Involved in Pathogen Appressoria Formation and Virulence Improves Tobacco Resistance to *Ciboria shiraiana* .” Journal of Fungi 7: 1053. 34947035 10.3390/jof7121053 PMC8709418

[mpp70209-bib-0058] Zhu, X. M. , L. Li , M. Wu , et al. 2019. “Current Opinions on Autophagy in Pathogenicity of Fungi.” Virulence 10, no. 1: 481–489. 30475080 10.1080/21505594.2018.1551011 PMC6550554

