# Supplementary material for: Regulator of G‐Protein Signalling Protein AaRgs2 Negatively Regulates Appressorium‐Like Formation of Alternaria alternata Induced by Pear Cutin Monomer via the AaRgs2‐AaGα1‐AaAC Module
Source: Mol Plant Pathol. 2026 Jan 23;27(1):e70209. doi: 10.1111/mpp.70209 (PMC12830874; doi:10.1111/mpp.70209)
Supplement: Supplementary file 1 — Figure S1: mpp70209‐sup‐0001‐FigureS1.pptx. [file MPP-27-e70209-s005.pptx]

## Slide 1
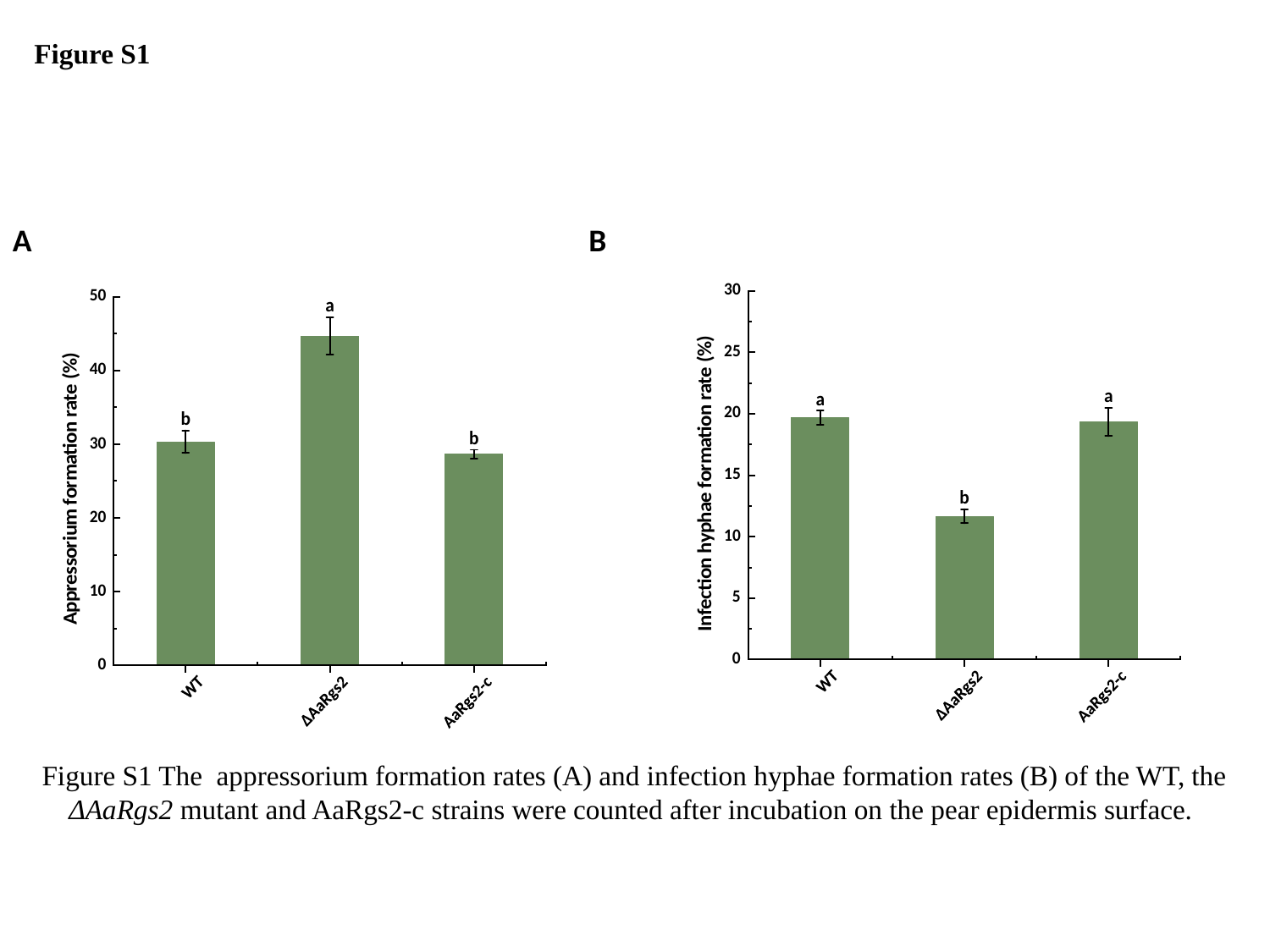

Figure S1
A
B
Figure S1 The appressorium formation rates (A) and infection hyphae formation rates (B) of the WT, the ΔAaRgs2 mutant and AaRgs2-c strains were counted after incubation on the pear epidermis surface.
