# Supplementary figures and images for: Regulator of G‐Protein Signalling Protein AaRgs2 Negatively Regulates Appressorium‐Like Formation of Alternaria alternata Induced by Pear Cutin Monomer via the AaRgs2‐AaGα1‐AaAC Module
Source: Mol Plant Pathol. 2026 Jan 23;27(1):e70209. doi: 10.1111/mpp.70209 (PMC12830874; doi:10.1111/mpp.70209)

## Slide 1
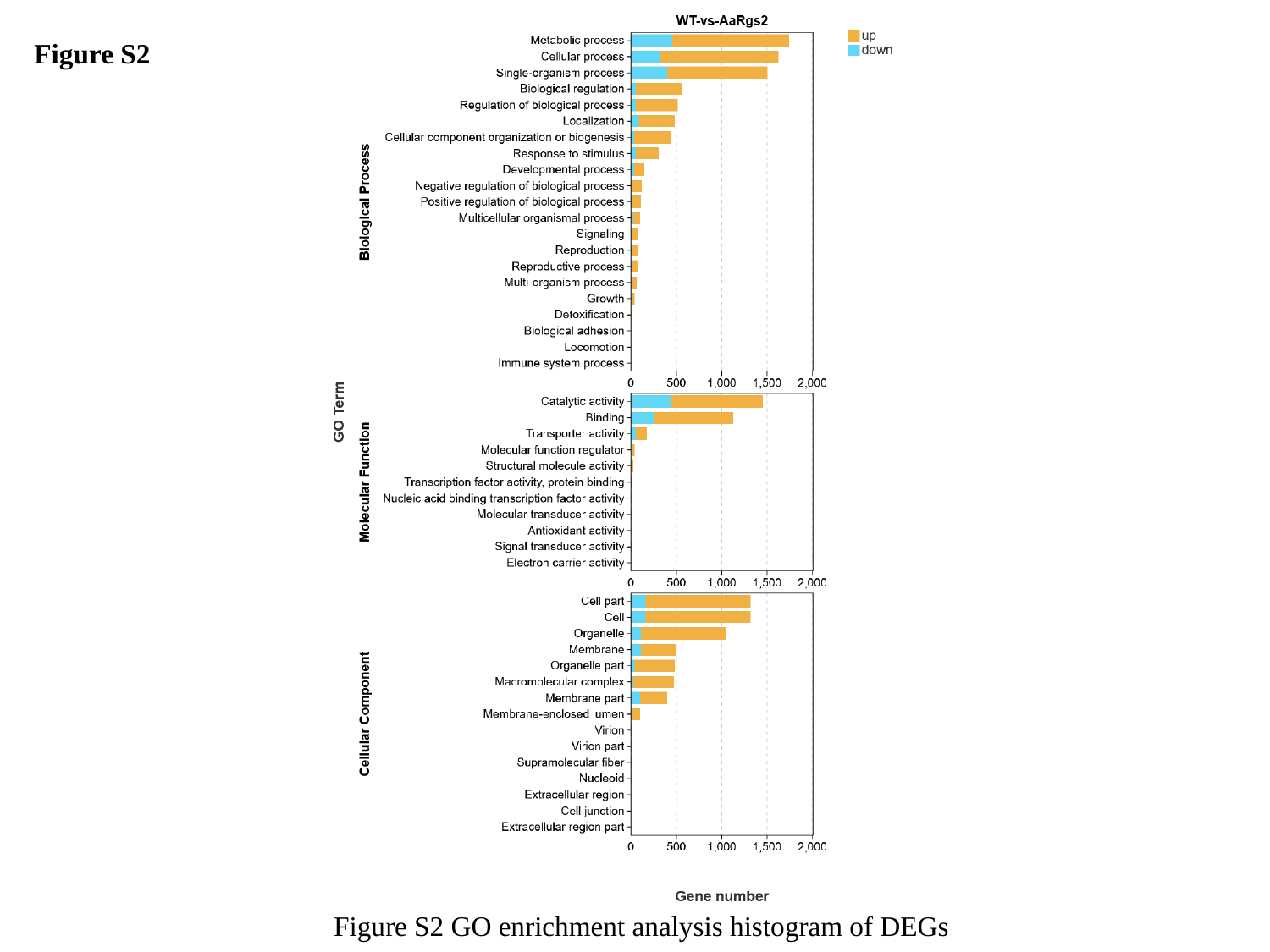

Figure S2
Figure S2 GO enrichment analysis histogram of DEGs

Supplement: Supplementary file 2 — Figure S2: mpp70209‐sup‐0002‐FigureS2.pptx. [file MPP-27-e70209-s003.pptx]

## Slide 1
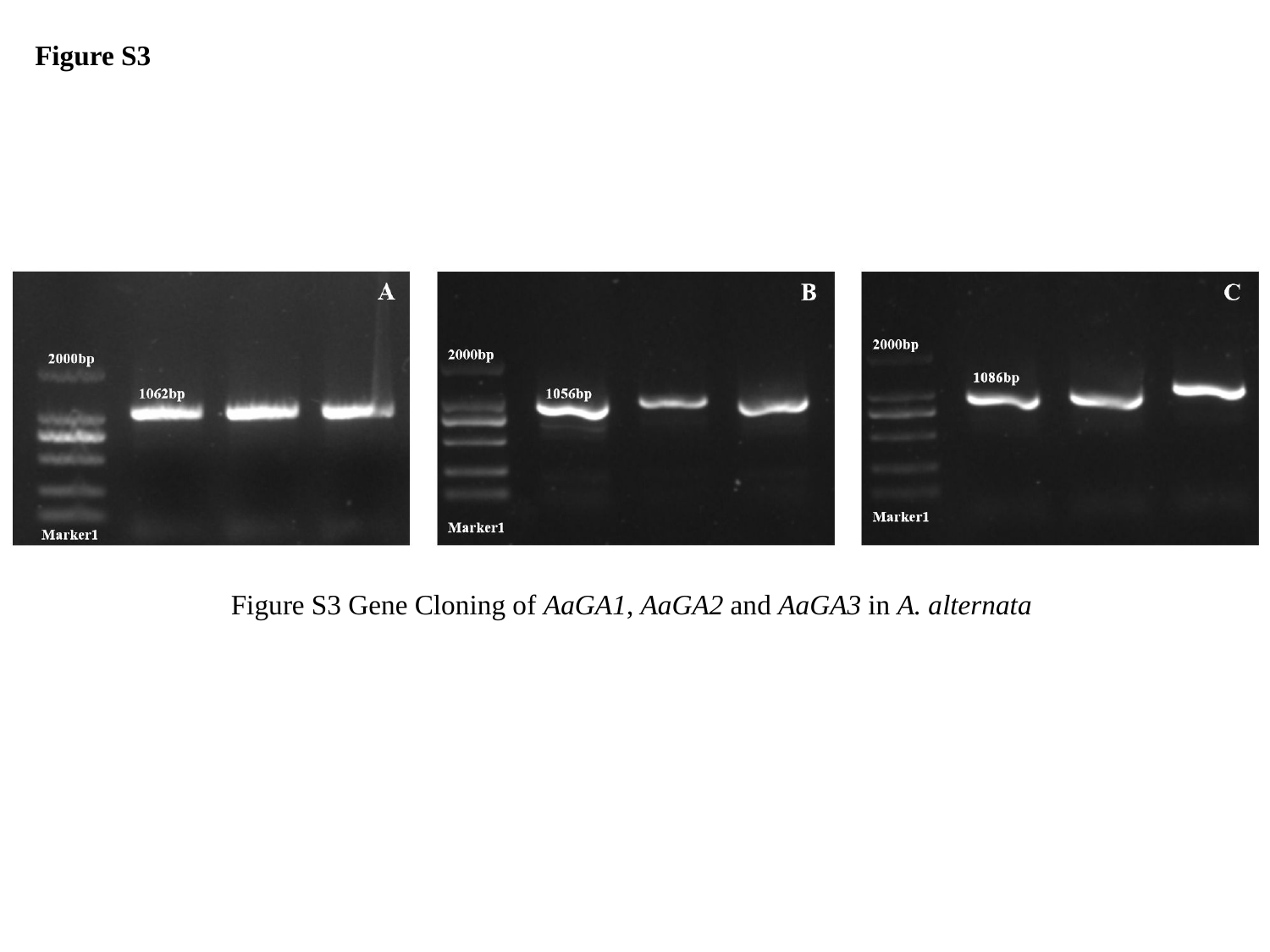

Figure S3
Figure S3 Gene Cloning of AaGA1, AaGA2 and AaGA3 in A. alternata

Supplement: Supplementary file 3 — Figure S3: mpp70209‐sup‐0003‐FigureS3.pptx. [file MPP-27-e70209-s001.pptx]
