# Supplementary material for: Regulator of G‐Protein Signalling Protein AaRgs2 Negatively Regulates Appressorium‐Like Formation of Alternaria alternata Induced by Pear Cutin Monomer via the AaRgs2‐AaGα1‐AaAC Module
Source: Mol Plant Pathol. 2026 Jan 23;27(1):e70209. doi: 10.1111/mpp.70209 (PMC12830874; doi:10.1111/mpp.70209)
Supplement: Supplementary file 4 — Figure S4: mpp70209‐sup‐0004‐FigureS4.pptx. [file MPP-27-e70209-s004.pptx]

## Slide 1
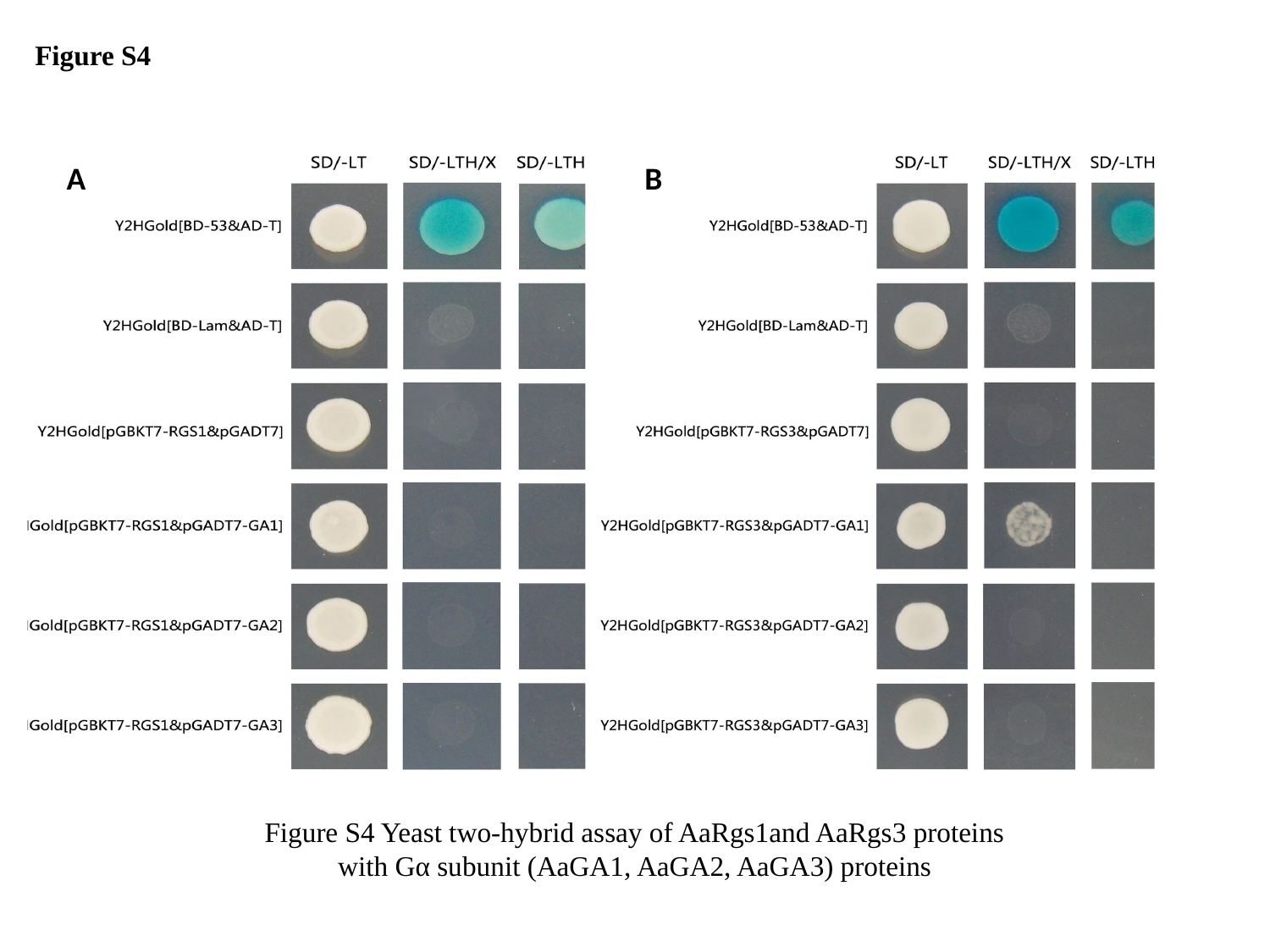

Figure S4
A
B
Figure S4 Yeast two-hybrid assay of AaRgs1and AaRgs3 proteins
with Gα subunit (AaGA1, AaGA2, AaGA3) proteins
