# Supplementary material for: Regulator of G‐Protein Signalling Protein AaRgs2 Negatively Regulates Appressorium‐Like Formation of Alternaria alternata Induced by Pear Cutin Monomer via the AaRgs2‐AaGα1‐AaAC Module
Source: Mol Plant Pathol. 2026 Jan 23;27(1):e70209. doi: 10.1111/mpp.70209 (PMC12830874; doi:10.1111/mpp.70209)
Supplement: Supplementary file 5 — Table S1: mpp70209‐sup‐0005‐TableS1.docx. [file MPP-27-e70209-s006.docx]

**Table S1 qRT-PCR primers used in this study**

| Primer | Sequence | Characteristic(s) |
| --- | --- | --- |
| *AC*-F | 5´-AGTACGAAAGAGTTGCCTCG-3´ | *AC* gene expression level |
| *AC*-R | 5´-GAAGTCAAATGTTTTGAAAT-3´ |  |
| *PdeL*-F | 5´-AGGAACATCAAAATGGCACG-3´ | *PdeL* gene expression level |
| *PdeL*-F | 5´-GCGTCAAAAGCAGCAGATAGTA-3´ |  |
| *STE11*-F | 5´-CCTCGCTATCTGAACGCTTGA-3´ | *STE11* gene expression level |
| *STE11*-R | 5´-GTCGATTCGCATCCTTGACA-3´ |  |
| *Cdc42*-F | 5´-CCCCAAACCGACGTCTTTC-3´ | *Cdc42* gene expression level |
| *Cdc42*-R | 5´-AAAGGAAGCGGGCGAAGTT-3´ |  |
| *Fus3*-F | 5´-TCGAGCGGTTACTCGCTTTC-3´ | *Fus3* gene expression level |
| *Fus3*-R | 5´-TGAGCGCTTCCTCGACTGT-3´ |  |
| *GNB1*-F | 5´-ACGCGTAGGCACATTGCAA-3´ | *GNB1* gene expression level |
| *GNB1*-R | 5´-TTGCTGACACCGAGACAACTG-3´ |  |
| *SteA*-F | 5´-TGCTGAGCCTGCCTACATTG-3´ | *SteA* gene expression level |
| *SteA*-R | 5´-GGTCTGGAATGGCGCTGTAA-3´ |  |
| *Ste7*-F | 5´-GAACGATCTCCTCCCCACAA-3´ | *Ste7* gene expression level |
| *Ste7*-R | 5´-CGCCGTTGGCTACTCTTTG-3´ |  |
| *ATG1*-F | 5´-CGCAAACACAACGAGATCGT-3´ | *ATG1* gene expression level |
| *ATG1*-R | 5´-GTTGTCGAACGTCGCTTCATT-3´ |  |
| *ATG13*-F | 5´-CAAAAAGGCCGTGGTCACA-3´ | *ATG13* gene expression level |
| *ATG13*-R | 5´-GCGGGCATGAAGCGTAGATA-3´ |  |
| *VPS15*-F | 5´-CGAACTCCCCACTCGCTTT-3´ | *VPS15* gene expression level |
| *VPS15*-R | 5´-TTCCTAAATCGGATCCGCATA-3´ |  |
| *VPS34*-F | 5´-TCCCATCGATGAGCATAGCA-3´ | *VPS34* gene expression level |
| *VPS34-*R | 5´-AGGCGAGTAGCGAACCTTTG-3´ |  |
| *ATG10*-F | 5´-GCAGGATCGAGACGCTACTGA-3´ | *ATG10* gene expression level |
| *ATG10-*R | 5´-CTACGGTGGTGTCGAGAGCTT-3´ |  |
| *ATG7*-F | 5´-TCCCATCGATGAGCATAGCA-3´ | *ATG7* gene expression level |
| *ATG7-*R | 5´-AGGCGAGTAGCGAACCTTTG-3´ |  |
| *PEX19*-F | 5´-CTTGACGATGTGCTCGATGAG-3´ | *PEX19* gene expression level |
| PEX19-R | 5´-ACGAAGCGGTAGGAGCTTTG-3´ |  |
| *PEX1*-F | 5´-CACGCGTCGTTGCTAAGATC-3´ | *PEX1* gene expression level |
| *PEX1*-R | 5´-AGGGCTACATGCCGACTGTCT-3´ |  |
| *PEX6*-F | 5´-CCGCTTCAAAATCGCTTACC-3´ | *PEX6* gene expression level |
| *PEX6*-R | 5´-CCGCCGGCTAGTTCAATGT-3´ |  |
| *PEX10*-F | 5´-CACAGGAGAATGGGAGCAAAG-3´ | *PEX10* gene expression level |
| *PEX10*-R | 5´-GTTTGCAGGCGCATACCAA-3´ |  |
| *PEX2*-F | 5´-TCACCGAGAGCTCCGAATG-3´ | *PEX2* gene expression level |
| *PEX2*-R | 5´-TGCTCATCATCACTGCTTCGA-3´ |  |
| *GAPDH*-F | 5´-ATTGTCGCCGTAAACGACCC-3´ | Reference gene expression level |
| *GAPDH*-R | 5´-TTGACGGTCAGGTTGTTGCC-3´ |  |
