# Supplementary material for: Regulator of G‐Protein Signalling Protein AaRgs2 Negatively Regulates Appressorium‐Like Formation of Alternaria alternata Induced by Pear Cutin Monomer via the AaRgs2‐AaGα1‐AaAC Module
Source: Mol Plant Pathol. 2026 Jan 23;27(1):e70209. doi: 10.1111/mpp.70209 (PMC12830874; doi:10.1111/mpp.70209)
Supplement: Supplementary file 6 — Table S2: mpp70209‐sup‐0006‐TableS2.docx. [file MPP-27-e70209-s002.docx]

**Table S2 Yeast two-hybrid primers used in this study**

| Primer | Sequence |
| --- | --- |
| pGBKT7-AaRgs2-F | 5´-ATATGGCCATGGAGGCCGAATTCCTGGCGCAACAACTCCAACT-3´ |
| pGBKT7-AaRgs2-R | 5´- TTATGCGGCCGCTGCAGGTCGACTTAGAAGAGACCACCCTCAA-3´ |
| pGADT7-AaGA1-F | 5´-TGGCCATGGAGGCCAGTGAATTCATGGGTTGCGGAATGAGCAC-3´ |
| pGADT7-AaGA1-R | 5´-CTACGATTCATCTGCAGCTCGAGTTATATCAAACCGCACAACCG-3´ |
| pGADT7-AaGA2-F | 5´-TGGCCATGGAGGCCAGTGAATTCATGAGCACCAACAATGACGA-3´ |
| pGADT7-AaGA2-R | 5´-CTACGATTCATCTGCAGCTCGAGTCATAGTATGCCGGAGTCTTT-3´ |
| pGADT7-AaGA3-F | 5´-TGGCCATGGAGGCCAGTGAATTCATGGCACCGGCAATAATGTGC-3´ |
| pGADT7-AaGA3-R | 5´-CTACGATTCATCTGCAGCTCGAGTTATAGTATCAGGGCGTTAAG-3´ |
| pGBKT7-pfam-F | 5´-TGGCCATGGAGGCCAGTGAATTCATGACGCGGAATGAGACCAT-3´ |
| pGBKT7-pfam-R | 5´-CTACGATTCATCTGCAGCTCGAGTCAGCGATCCTGATCGGTGTAT-3´ |
| pGBKT7-PP2C-F | 5´-TGGCCATGGAGGCCAGTGAATTCATGGTGCGCACTGCTGGCTCG-3´ |
| pGBKT7-PP2C-R | 5´-CTACGATTCATCTGCAGCTCGAGTCAGCCTTCAATGTGCGTCTC-3´ |
| T7-F | 5´-TAATACGACTCACTATAGGGC-3´ |
| 3’AD-R | 5´-AGATGGTGCACGATGCACAG-3´ |
| 3’BD-R | 5´-TTTTCGTTTTAAAACCTAAGAGTC-3´ |

Note: The underlined portion indicates the restriction enzyme site.
